# Blueberry Attenuates Liver Fibrosis, Protects Intestinal Epithelial Barrier, and Maintains Gut Microbiota Homeostasis

**DOI:** 10.1155/2019/5236149

**Published:** 2019-11-22

**Authors:** Zhiqiang Yan, Fang Yang, Zhu Hong, Shishun Wang, Zhang Jinjuan, Bing Han, Rujia Xie, Feiyun Leng, Qin Yang

**Affiliations:** ^1^Department of Pathophysiology, Basic Medical College, Guizhou Medical University, Guiyang, Guizhou 550004, China; ^2^Department of Gastrointestinal Surgery, The Affiliated Hospital of Guizhou Medical University, Guiyang, Guizhou 550004, China; ^3^Guizhou Provincial Key Laboratory of Pathogenesis & Drug Research on Common Chronic Diseases, Guizhou Medical University, Guiyang, Guizhou 550004, China; ^4^Department of Clinical Hematology, College of Medical Laboratory, Guizhou Medical University, Guiyang, Guizhou 550004, China; ^5^Department of Pathology, Qingzhen People's Hospital, Guiyang, Guizhou 550004, China; ^6^Functional Laboratory of Basic Medical College of Guizhou Medical University, Guiyang, Guizhou 550004, China; ^7^Department of Pathology, The First People's Hospital of Guiyang, Guiyang, Guizhou 550004, China

## Abstract

**Objective:**

Recently, blueberry has been identified as a candidate for the treatment of liver fibrosis. Given the role of gut-liver axis in liver fibrosis and the importance of the gut microbiota homeostasis to the maintenance of the intestinal epithelial barrier, this study aimed to investigate whether blueberry could attenuate liver fibrosis and protect the intestinal epithelial barrier by maintaining the homeostasis of the gut microbiota.

**Method:**

A CCl_4_-induced rat liver fibrosis model was used to detect the roles of blueberry in liver fibrosis and intestinal epithelial barrier. The liver weight and body weight were measured, the liver function was monitored by ALT and AST activity, protein and mRNA were determined by western blot and RT-qPCR, and the gut microbiome was detected by Miseq.

**Results:**

The results showed that blueberry could reduce the rate of liver weight/body weight gain (*p* < 0.05), ALT (*p* < 0.01) and AST (*p* < 0.05) activity, and the expression of collagen I (*p* < 0.01), collagen IV (*p* < 0.01), and *α*-SMA (*p* < 0.01) expression in CCl_4_-induced rat liver. CCl_4_ impaired the intestinal epithelial barrier and decreased the expression of the tight junction protein. Blueberry restored the intestinal epithelial barrier and increased the expression of the tight junction protein. The gut microbiota homeostasis was impaired by CCl_4_, but after treatment with blueberry, the intestinal flora returned to normal.

**Conclusion:**

Blueberry attenuated liver fibrosis, protected intestinal epithelial barrier, and maintained the homeostasis of the gut microbiota in a CCl_4_-induced injury rat model.

## 1. Introduction

Liver fibrosis refers to the excessive accumulation of extracellular matrix (ECM) proteins, including hepatic collagen, which results from chronic liver injury and occurs in most chronic liver diseases [1]. The main causes of liver fibrosis include chronic HCV/HBV infection, alcohol abuse, and nonalcoholic steatohepatitis (NASH) [1]. Advanced liver fibrosis leads to liver cirrhosis, resulting in portal hypertension and liver failure and often requires liver transplantation. Liver fibrosis is also a key risk factor for hepatocellular carcinoma progression [2]. The liver injury model induced by CCl_4_ has been widely used in the study of liver fibrosis, liver necrosis, and medicinal plant extract assessments [3, 4]. Continuous CCl_4_ treatment increases hepatic cell damage and results in pathological processes of hepatocytes, which undergo apoptosis and regeneration and gradually return to normal. However, when necrotic liver cells exceed their regenerative capacity, they can lead to liver cell damage and even liver failure [5].

The intestinal tract and the liver are anatomically and physiologically connected. The relationship between the two has been called the “gut-liver axis,” and the effects of intestinal metabolites on the liver are essential to the onset and progression of liver diseases [6–8]. The gut microbiota, in particular, has recently emerged as an important gut-liver axis-mediated factor [9–11]. Alterations of the intestinal microbial composition could induce liver damage and initiate liver fibrosis changes to the development of cirrhosis and associated complications [12]. The tight junctions (TJ) within the gut epithelium represent a natural barrier to bacteria and their metabolic products [13]. Breaking down the barrier allows harmful components to injure the liver. Therefore, the gut-liver axis is an important focus for the treatment of liver diseases.

Blueberry (BB) is a flowering plant that belongs to *Vaccinium* spp. of the family Ericaceae. The Human Nutrition Research Center (Mayer, USA) has conducted a series of in-depth studies on blueberries. These studies have indicated that blueberries contain anthocyanins, polyphenols, and flavonoids and appear to have the highest antioxidant capacity among common fruits and vegetables [14, 15]. Blueberries may also have anti-inflammatory and antitumor effects [16, 17]. Recently, BB has been found to be a potential candidate for the treatment of liver fibrosis [18–21]. However, the underlying mechanisms remain unclear.

In this study, we aimed to investigate the preventative effects of BB treatment on CCl_4_-induced liver fibrosis, intestinal epithelial barrier disruption, and gut microbiota imbalance. The results demonstrated that treatment with BB improved liver fibrosis, intestinal epithelial barrier balance, and gut microbiota homeostasis.

## 2. Materials and Methods

### 2.1. Chemicals and Materials

CCl_4_ and other chemicals were supplied by Chengdu Jinshan Chemical Reagent Co., Ltd. CCl_4_ was dissolved in vegetable oil.

### 2.2. Animals and Experimental Design

Male Sprague-Dawley rats weighing 200–250 g were selected for use in the study. They were obtained from the Laboratory Animal Center of the Third Military Medical University of the People's Liberation Army. Rats were housed in a light- and temperature-controlled room on a 12/12 h light/dark cycle. The animals were allowed free access to food and water and were kept in SPF. The whole experiment was conducted in accordance with the guidelines of the Animal Care and Use Committee of Guizhou Medical University.

Rats were randomly divided into two groups as follows: Ctrl group and CCl_4_ group. The rats in the Ctrl group were injected with vehicle every other day for 8 weeks, while the rats in the CCl_4_ group were injected every other day with CCl_4_ (3 ml/kg 40% CCl_4_; i.p.) for 8 weeks. The Ctrl group was randomly subdivided into two groups as follows: (1) Ctrl (control) group (*n* = 6): animals were fed with 10 ml/kg saline daily by gavage and (2) BB (blueberry) group (*n* = 6): rats were perfused daily with 10 ml/kg BB juice by gavage. The CCl_4_ group was also randomly subdivided into two groups: (3) CCl_4_ (*n* = 6): rats were injected with CCl_4_ (3 ml/kg 40% CCl_4_; i.p.) once a week, and 10 ml/kg saline was administered daily by gavage and (4) CCl_4_ + BB group (*n* = 6): rats were injected with CCl_4_ (3 ml/kg 40% CCl_4_; i.p.) once a week, and 10 ml/kg BB juice was administered daily by gavage. The rats were fed for another 8 weeks for a total of 16 weeks.

### 2.3. Blood and Tissue Samples

At the end of the treatment, all rats were sacrificed by cardiac puncture under sodium thiopental anesthesia (50 mg/kg, i.p.). Blood was collected in dry tubes. The livers and colon tissues were rapidly removed, washed in 0.9% NaCl, and stored in ice. The materials were stored at −80°C until the main analysis.

### 2.4. Determinations in Serum

Alanine aminotransferase (ALT) and aspartate aminotransferase (AST) measurements were performed on a Siemens ADVIA-2400 automatic biochemical analyzer.

### 2.5. Western Blot Analysis

Total protein was extracted from the rat liver and colon with RIPA buffer. Protein concentration was determined via BCA assay. Equal amounts of protein and 5x SDS loading buffer were mixed and boiled for 5 min. Proteins (30 *μ*g per lane) were separated via SDS-PAGE on a 10% gel and transferred onto polyvinylidene difluoride membranes (Zhongshan Jinqiao Biology and Technology Co., Ltd., Beijing, China). Membranes were blocked with 5% nonfat milk for 1 h at room temperature. The membranes were incubated with primary antibodies against Collagen I (3241980-1, Abcam), Collagen IV (AA06195689, Bioss), *α*-SMA (GR282976-35, Abcam), Claudin1 (ab15098, Abcam), Claudin2 (ab53032, Abcam), ZO1 (21773-1-AP, Proteintech Group, Inc), and *β*-actin overnight at 4°C, respectively. Following primary incubation, membranes were incubated with horseradish peroxidase-labeled goat antimouse or antirabbit immunoglobulin G secondary antibody (A0208, Beyotime Biotechnology) for 1 h at room temperature. Protein bands were visualized using an ECL Prime Western Blotting Detection Reagent (RPN2232SK, Shanghai Haoran Biotechnology Co., Ltd.).

### 2.6. Reverse Transcription-Quantitative Polymerase Chain Reaction (RT-qPCR)

Total RNA was extracted from the colon using TRIzol reagent (TAKARA). Total RNA (2 *μ*g) was reverse transcribed into cDNA using the BestarTM qPCR RT kit (DBI). To detect the mRNA expression levels of cluadin1, cluadin2, and ZO1, qPCR was performed using the BestarTM qPCR MasterMix (DBI) on an ABI 7900 Real-Time PCR system (Applied Biosystems; Thermo Fisher Scientific, Inc.). The following primer pairs were used for qPCR: claudin1: forward, 5′-GGACAACATCG TGACTGCTC-3′ and reverse, 5′-CCCAGCAGG ATGCCAATTAC-3′; claudin2: forward, 5′-GCTGTAGTGG GTGGAGTCTT-3′ and reverse, 5′-GGCCTGGTAG CCATCATAGT-3′; ZO1: forward, 5′-CACCTCGCACG TATCACAAG-3′ and reverse, 5′-GGCAATGACAC TCCTTCGTC-3′; GAPDH: forward, 5′-CCTCGTCTCAT AGACAAGATGGT-3′ and reverse, 5′-GGGTAGAGTCATA CTGGAACATG-3′. The thermocycling conditions for qPCR were as follows: initial denaturation at 95°C for 10 sec and 40 cycles of 95°C for 10 sec and 60°C for 60 sec. The expression was quantified using the 2^−ΔΔ^Cq method and normalized to the internal reference gene GAPDH.

### 2.7. Histological Analysis

The liver and colon tissues were paraffin-embedded after fixation and cut into 5 *μ*m thick sections. Afterwards, the haematoxylin and eosin (H&E) stain was used to stain sections of liver and colon for histological analysis according to standard instructions. According to the standard instructions, Masson's stain was also performed, with the results showing reticulin fibers in the fibrotic areas. In order to randomly select microscopic areas in liver sections for examination, a Ti-S inverted fluorescence microscope (Nikon, Tokyo, Japan) was used.

### 2.8. 16S rRNA Gene V4 Amplification, Quantitation, and Sequencing

The sample was subjected to targeted metagenomic analysis by sequencing the V4 region of the 16S rRNA gene. Primers modified from Caporaso et al. [22] were utilized to conduct V4 amplification. As a brief introduction, these primers were designed to amplify the 16S rRNA gene from 515 to 806, which includes a barcode and an adapter for annealing to the Illumina flow cell. The primers in this study differed from those in Caporaso et al. because both of the primers contained a 12 bp barcode and not just the reverse primer (Dataset S25). This makes it possible to pool many samples using a unique combination of barcodes, overcoming the dependence on a large number of reverse primers with unique barcodes. The PCR reaction mixture was prepared using Qiagen HotStar HiFidelity polymerase. Qiagen HotStar HiFidelity polymerase was employed to prepare the PCR reaction mixture. Each mixture had a volume of 25 *μ*L and was polymerized using 0.5 *μ*L HotStar 92 polymerase, 1 *μ*L sample DNA, 2.5 *μ*L reverse primer (10 *μ*M), 2.5 91 *μ*L forward primer (10 *μ*M), 5 *μ*L HotStar PCR Buffer, and 14 *μ*L·H_2_O. A touchdown PCR program was utilized on a Biometra TProfessional Basic Gradient thermocycler: 95°C for 5 min, followed by 7 cycles of 95°C for 45 sec, 65°C for 1 min (decreasing at 2°C/94 cycle), and 72°C for 90 sec, followed by 30 cycles of 95°C for 45 sec, 50°C for 30 sec, and 72°C for 95 sec. A final extension at 72°C was carried out for 10 min, and the reactions were held at 4°C. The reactions were run on a 1% agarose gel in order to ensure the success of the amplification. Unsuccessful reactions were followed by one more trial, failure of which would lead to removal from the experiment. The amplicon libraries were diluted 40x. To quantify the amplicons, either an Agilent Bioanalyzer (greenhouse libraries) or a Caliper LabChip GX (field experiment libraries) was used at the DNA Technologies Core at the Genome Center, UC Davis. The libraries were then pooled into 4 pooled libraries (2 for the greenhouse experiment and 2 for the field experiment) at equivalent concentrations. To remove any primer dimer from the polymerized amplicon libraries, the 4 pooled libraries were run on 1.8% 5 agarose gels and a 400 bp band was extracted. As a final check of quality, the bands were subjected to purification (Macherey-Nagel Nucleospic Gel and 104 PCR Cleanup kit) and bioanalysis. Afterwards, each library was submitted to the UC Davis DNA Technologies core for 250 × 250 paired end and dual index sequencing on an Illumina MiSeq instrument.

### 2.9. Sequence Analysis

According to the barcode sequences, a customized Perl script based on the principle of exact matching was utilized to decompile the sequences obtained from the MiSeq runs. MOTHUR's command make.contigs was adopted to form contiguous reads by overlapping these sequences [23]. A read containing any base of ambiguity, along with any read exceeding 275 bp was then discarded. The sequences were then clustered into operational taxonomic units (OTUs) by UCLUST [24] based on 97% pairwise identity. This was performed using QIIME's [25] open reference OTU picking strategy based on the Greengenes 16S rRNA database (13_5 release) as a reference [26]. Using default parameters, the QIIME's version of the Ribosomal Database Project's classifier [27] was pitted against the Greengenes 16S rRNA database (13_5 release) to develop a taxonomy of the representative sequences for each OTU. All OTUs identified as belonging to chloroplast and mitochondria were deleted from the data set. PyNAST [28] in QIIME was adopted for alignment of the representative sequences for each OTU. QIIME's implementation of ChimeraSlayer [29] was performed to identify the Chimeric OTUs, which were then removed from the OTU table and OTU representative sequence file. FastTree [30] generated a phylogenetic tree from the alignment file.

### 2.10. Statistical Analysis

Data were reported as mean ± standard deviation. All statistical analyses were performed using GraphPad Prism (Version 8; GraphPad Software, Inc., La Jolla, CA, USA). The two-way ANOVA test was used to analyze differences among the four groups. Statistical significance was obtained at *p* < 0.05.

## 3. Results

### 3.1. Blueberry Improved CCl_4_-Induced Liver Weight Loss and Liver Function

**Figure 1:**
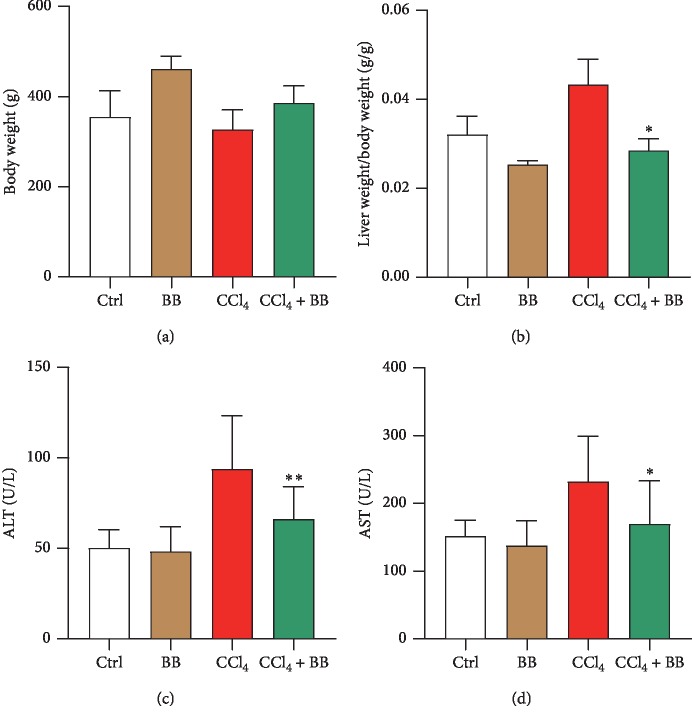
Blueberry attenuated CCl_4_-induced liver injury The body weights (a) and liver weights/body weights (b) in the Ctrl, BB, CCl_4_, and CCl_4_ + BB groups. The plasma levels of ALT (c) and AST (d) in the Ctrl, BB, CCl_4_, and CCl_4_ + BB groups. *n* = 6. ^*∗*^*p* < 0.05 and ^*∗∗*^*p* < 0.01.

To ascertain the effects of BB on CCl_4_-induced liver injury, rats were injected with CCl_4_ for 8 weeks and then subjected to treatment with BB by gavage for another 8 weeks. Throughout the entire process, the rats' body and liver weight were constantly monitored. The results demonstrated that no significant difference in body weight was found between the control group and the experimental group (Figure 1(a)). The administration of CCl_4_ increased liver weight, and treatment with BB prevented CCl_4_-induced liver weight gain (*p* < 0.05) (Figure 1(b)). A significant increase in the serum activities of ALT and AST, two reliable indicators of liver injury [21], is evidence of hepatocellular damage. Treatment with CCl_4_ indeed contributed to a significant increase in serum ALT and AST. It was observed that these enzyme activities decreased in CCl_4_-treated rats due to BB treatment (Figures 1(c) and 1(d)). The differences of ALT and AST were statistically significant (*p* < 0.01 for ALT, *p* < 0.05 for AST). These data indicated that blueberry could improve CCl_4_-induced liver damage.

### 3.2. Blueberry Attenuated CCl_4_-Induced Liver Fibrosis

**Figure 2:**
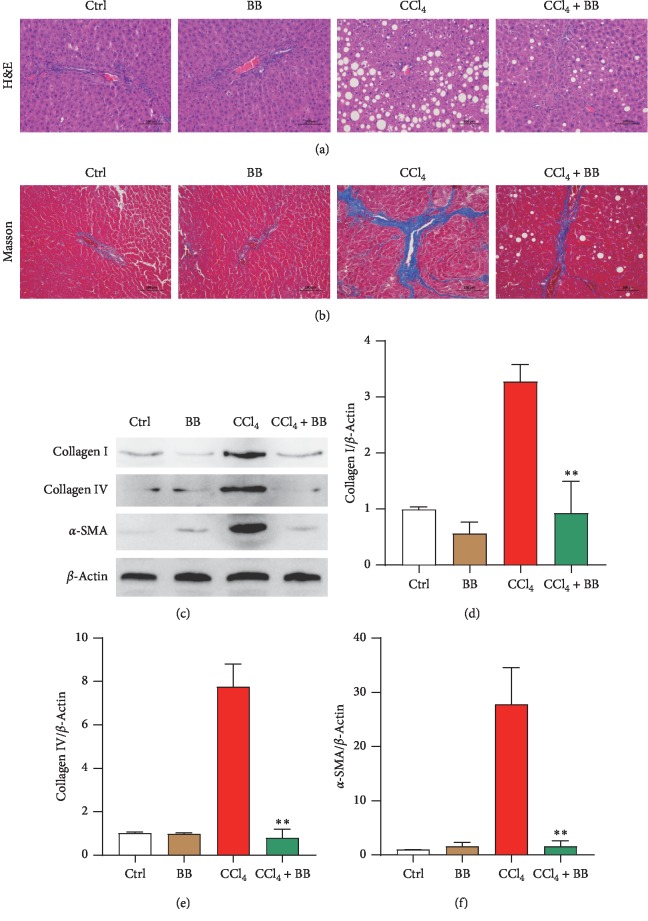
Blueberry attenuated CCl_4_-induced liver fibrosis. H&E (a) and Masson's (b) staining in rat livers among the Ctrl, BB, CCl_4_, and CCl_4_ + BB groups. (c–f) The protein levels of collagen I, collagen IV, and *α*-SMA in rat livers among the Ctrl, BB, CCl_4_, and CCl_4_ + BB groups. Western blot was performed to identify the protein levels. (c) The bands of collagen I, collagen IV, and *α*-SMA. (d–f) Quantitative analysis of collagen I (d), collagen IV (e), and *α*-SMA (f). *β*-Actin acted as a loading control (*n* = 6, ^*∗∗*^*p* < 0.01).

Then, the role of BB in CCl_4_-induced liver fibrosis was examined. After HE and Masson's staining, there were signs of CCl_4_-induced liver collagen fiber regeneration, diffuse steatosis, hepatic plate disorder, and lobular structure destruction (Figures 2(a) and 2(b)). Treatment with BB improved the dysfunction induced by CCl_4_ (Figures 2(a) and 2(b)). Western blot was conducted in each group to detect the fibrotic protein collagen I, collagen IV, and *α*-SMA. The results showed that treatment with BB reduced the expression of collagen I, collagen IV, and *α*-SMA induced by CCl_4_ (Figures 2(c)–2(f)). The differences were statistically significant (*p* < 0.01). These data also indicated that BB could alleviate CCl_4_-induced liver fibrosis.

### 3.3. Blueberry Protected Intestinal Epithelial Barrier Breakdown Induced by CCl_4_

**Figure 3:**
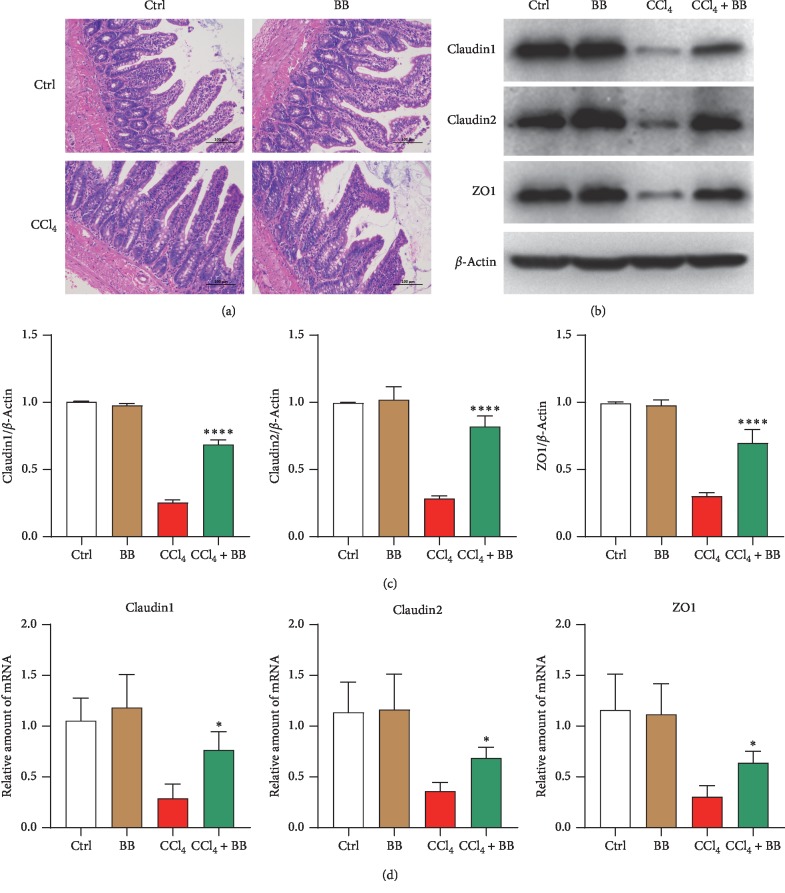
Blueberry protected intestinal epithelial barrier breakdown induced by CCl_4_. (a) H&E staining in rat colon among the Ctrl, BB, CCl_4_, and CCl_4_ + BB groups. The protein bands (b) and quantitative value (c) of claudin1, claudin2, and ZO1 in rat colon among the Ctrl, BB, CCl_4_, and CCl_4_ + BB groups. *β*-Actin acted as a loading control. (d) The mRNA levels of claudin1, claudin2, and ZO1 in rat colon among the Ctrl, BB, CCl_4_, and CCl_4_ + BB groups. GAPDH acted as a loading control (*n* = 6, ^*∗*^*p* < 0.05 and ^*∗∗∗∗*^*p* < 0.0001).

The tight junctions (TJ) within the gut epithelium represent a natural barrier to bacteria and their metabolic products and play a key protective role in preventing liver injury [13]. Then, we tested whether BB could mitigate liver damage by maintaining the balance of the intestinal epithelial barrier. CCl_4_ disrupted the intestinal epithelial barrier and reduced the expressions of TJ protein (Figure 3). Treatment with BB could improve the structure of colon (Figure 3(a)) and increase the protein (Figures 3(b) and 3(c)) and mRNA levels (Figure 3(d)) of claudin1, claudin2, and ZO1 reduced by CCl_4_. The differences were statistically significant (*p* < 0.0001 for protein and *p* < 0.05 for RNA). These data indicated that BB could improve the intestinal epithelial barrier.

### 3.4. Effects of BB on the Alterations of the Gut Microbiota Induced by CCl_4_

**Figure 4:**
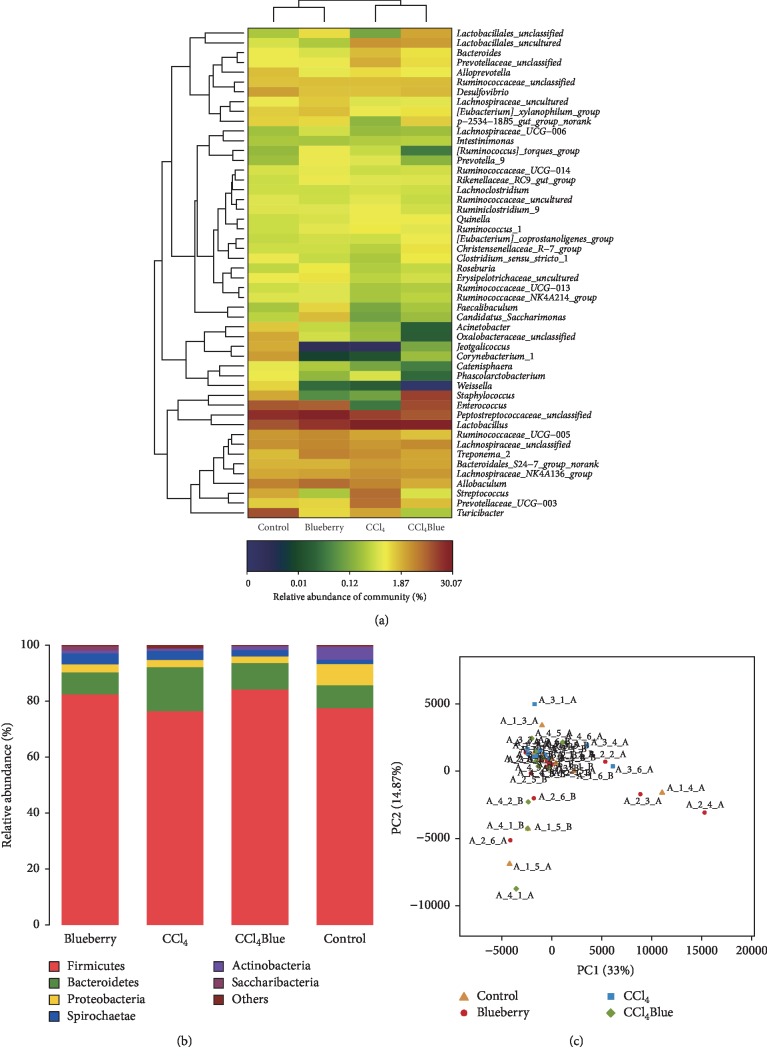
The heatmap (a), relative abundance (b), and PCA (c) of gut microbiota at the phylum level in the Ctrl, BB, CCl_4_, and CCl_4_ + BB groups.

An aggregate of 1,934,046 sequences was procured from the 24 faecal samples, with an average of 438.86 bp per sample. A total of 1352 OTUs (similarity greater than 97%) were determined after quality and chemical composition analysis. As Figures 4(a)–4(c) suggest, a significant decrease in firmicutes and bacteroidetes was observed following CCL_4_ treatment in the dominant phyla of the identified bacterial phyla, including *Bacillus* and Bacteroides. After BB treatment, the gut microbiota showed a recovery trend, suggesting that administration of BB helped prevent CCL_4_-induced hardness and changes in the number of bacteroides. After treatment with BB, the gut microbiome showed a trend of recovery, suggesting that BB administration contributed to the prevention of CCl_4_-induced alterations in the abundance of firmicutes and bacteroidetes.

**Figure 5:**
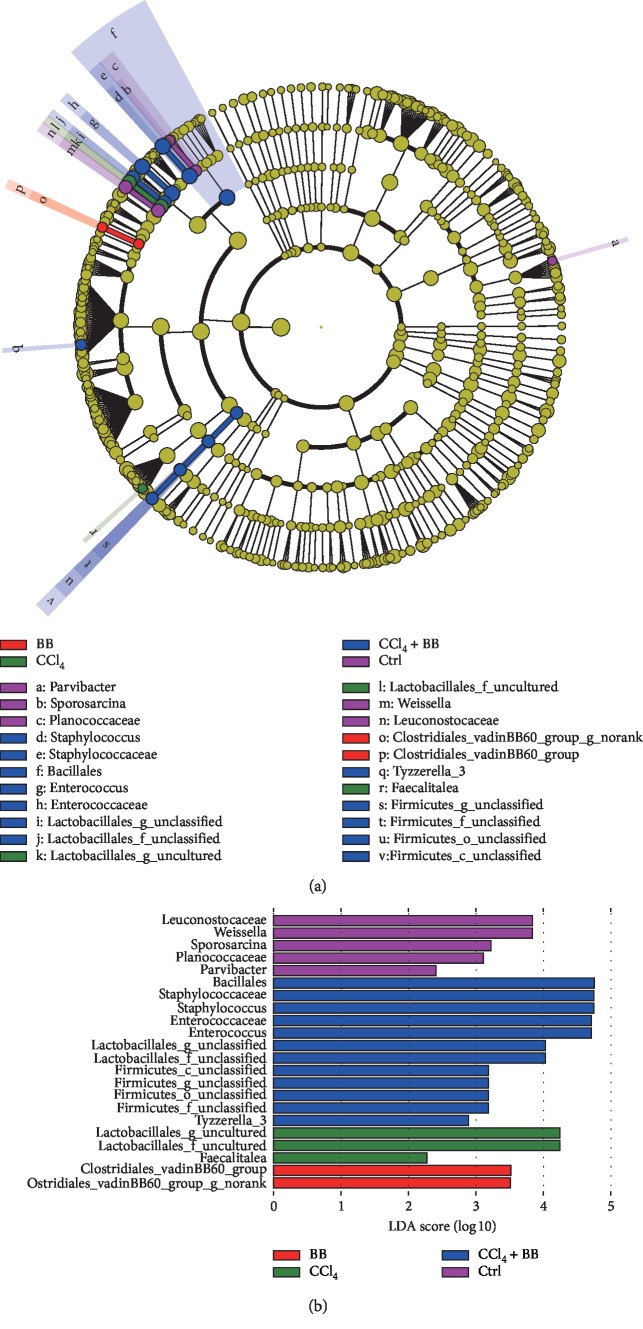
Differences in microbiota composition among the Ctrl, BB, CCl_4_, and CCl_4_ + BB groups with linear discriminant analysis effect size (LEFSE), visualized by cladogram (a) and histogram (b).

In order to compare the differences between groups with differing taxon abundance, a linear discriminant analysis effect size (LEFSE) method was adopted, with a higher score indicating greater consistency. Low-density lipoprotein A showed different taxa in the gut microbiota between different groups. LEFSE analysis was performed on the sequence of each sample to generate a cladogram. Through the cladogram, the different microbial communities of each group could be observed at different levels. Each circle represents the classification level from phylum to species. The diameter of the small circle is proportional to the relative abundance of gut microbiota. As shown in Figure 5, there were 22 largest differences in the intestinal flora (from phylum to species) of the four groups: the Ctrl group, the CCl_4_ group, the BB group, and the CCl_4_ + BB group (Figures 5(a) and 5(b)). These data suggest that oral administration of BB considerably contributed to the prevention of CCl_4_-induced alterations in the abundance of gut microbiota.

## 4. Discussion

The main cause of liver fibrosis is chronic liver injury, which is a reversible process in its early stage [31]. Previous studies have demonstrated that BB has a strong antiliver injury effect [18–21]. However, the underlying mechanisms of BB against liver fibrosis remain obscure. Therefore, this study sought to elucidate these mechanisms of BB in liver fibrosis. In this study, the liver fibrosis model was induced by CCl_4_ in rats. The serum ALT and AST enzymes' activities markedly increased after CCl_4_ administration, indicating serious hepatic injury; treatment with BB significantly decreased the activity of these enzymes (Figures 1(c) and 1(d)). The H&E and Masson's staining showed that CCl_4_ administration caused severe histological damage of liver tissue; however, treatment with BB notably attenuated the degree of liver injury (Figures 2(a) and 2(b)). These findings demonstrate that BB has a protective effect against CCl_4_-induced liver injury.

To ascertain the underlying mechanisms of BB in liver fibrosis, we first investigated the intestinal epithelial barrier. The intestinal epithelial barrier consists of multiple defense mechanisms, which can be divided into an epithelial (i.e., the mucus layer and epithelial cells) and an immunological barrier (i.e., the epithelial secretions and immune cells). In the current study, our principal focus was on the epithelial barrier, particularly the monolayer epithelial cells, which are interconnected by junctional complexes. On the one hand, the epithelium promotes the absorption of nutrients, water, and electrolytes in the lumen. On the other hand, it acts as a barrier preventing the transport of potentially harmful substances through the extracellular and paracellular compartment transport [32]. Paracellular transport is regulated by the apical junctional complex and consists of the tight junction (TJ) and the subjacent adherent junction (AJ) [33].

The TJs seal the paracellular space and form a selective barrier that allows transport via at least two pathways: a high-capacity, charge-selective pore pathway for small ions and uncharged molecules and a low-capacity leak pathway for larger molecules, regardless of charge [34]. TJs are considered highly dynamic, opening and closing continuously in response to various stimuli [35]. They consist of several transmembrane proteins, such as occludin, members of the claudin family, and junction adhesion molecules, as well as cytoplasmic plaque proteins, such as the zonula occludens proteins (i.e., ZO-1, ZO-2, and ZO-3), which connect the transmembrane proteins with the perijunctional actomyosin ring [33, 36]. Contraction of this ring is important in regulating paracellular permeability and is mainly mediated by activation of myosin light chain kinase (MLCK), which phosphorylates myosin II regulatory light chain (MLC) [33, 36]. In this study, we demonstrated that BB could improve the intestinal epithelial barrier broken down by CCl_4_ (Figure 3).

Now commonly referred to as “a new virtual metabolic organ,” the gut microbiota (GM) is a diverse ecosystem of archaea, bacteria, fungi, protozoa, and viruses that produces pathogenesis of the gastrointestinal tract, liver, respiratory, cardiovascular, endocrine, and many other diseases [37]. The gut-liver axis, which has received increasing attention in recent years, is the result of close anatomical and functional bidirectional interactions between the gastrointestinal tract and the liver, primarily through the portal vein [38]. The symbiotic relationship between GM and the liver is regulated and stabilized by a complex network of interactions, including metabolism, immunity, and neuroendocrine crosstalk between them [39]. Excessive intake of tissue-destructive foods, such as alcohol, CCl_4_, and/or high-fat diets (HFD), weakens the intestinal barrier function and produces large amounts of intestinal microbial components (so-called microbial-associated molecular patterns (MAMPs)), bacterial metabolites, and even intestinal microbiota, which are easy targets of transfer to the liver. As a consequence, this can lead to serious liver diseases, such as liver inflammation, fibrosis, and cancer [6]. Therefore, these intestinal microbial components and metabolites not only affect the intestinal tract where the gut microbes are located but also the organs that are located away from the gut through their systemic circulation [40, 41]. Our data demonstrated that BB could prevent CCl_4_-induced alterations in the abundance of gut microbiota (Figures 4 and 5).

In conclusion, the current study has demonstrated that BB prevented CCl_4_-induced gut microbiota alterations to attenuate liver fibrosis and maintain the balance of the intestinal epithelial barrier.

## Data Availability

The data used to support the findings of this study are available from the corresponding author upon request.
